# Advanced Eales’ Disease With Neovascular Glaucoma at First Presentation

**DOI:** 10.7759/cureus.18302

**Published:** 2021-09-26

**Authors:** Mohammed A Alfayyadh, Halla A Alabdulhadi, Mahdi H Almubarak

**Affiliations:** 1 Ophthalmology, Prince Mutaib bin Abdulaziz Hospital, Skaka, SAU; 2 Ophthalmology, Dhahran Eye Specialist Hospital, Dammam, SAU; 3 Ophthalmology, Dhahran Eye Specialist Hospital, Dhahran, SAU

**Keywords:** vasculitis, neovascular glaucoma, mycobacterium tuberculosis, eales’ disease, case report

## Abstract

Eales’ disease is an idiopathic vasculitis that affects the peripheral retina. It is characterized by recurrent vitreous hemorrhage as a complication of retinal neovascularization. It is more prevalent in India and affects young males. Here, we present a patient with neovascular glaucoma as a rare first presentation of Eales’ disease. This is a 24-year-old Indian male, who complained of a sudden decrease in vision in the left eye over less than 24 hours, along with frontal headache and eye pain for the last three weeks. Ocular examination revealed peripheral retinal ischemia in the right eye, very high intraocular pressure, rubeosis iridis, vitreous hemorrhage and extensive retinal ischemia in the left eye, vascular sheathing and neovascularization in both eyes. The purified protein derivative skin test was positive. The patient was managed with anti-glaucoma, intravitreal anti-vascular endothelial growth factor and laser photocoagulation. Systemic steroids and anti-tuberculous therapy were also initiated. Neovascular glaucoma is an infrequent complication of Eales’ disease. However, the lack of early detection of the disease in the early stages might lead to such serious complications.

## Introduction

Eales’ disease is an idiopathic peripheral obliterative vasculitis that mainly affects the peripheral retina [1]. It is characterized by the initial inflammatory stage represented by peripheral perivasculitis, followed by ischemic then proliferative stages [2]. Eales’ disease is broadly reported in Asia, particularly the Indian subcontinent, whereas it is rarely recorded in the western world. Moreover, it affects males more than females at 20-40 years of age [3]. Although a unilateral complaint is more frequent, 50%-90% of the patients have bilateral involvement [2,4]. The most prevalent etiological theory is hypersensitivity to tuberculin-protein that develops after exposure to Mycobacterium tuberculosis [5]. However, cases of active tuberculosis infection with concurrent Eales’ disease are unusual [6]. Complications of Eales’ disease include tractional retinal detachment, cataract, rubeosis iridis and phthisis bulbi. Moreover, neovascular glaucoma is a possible rare complication that might develop in the late stage of the disease [1].

To our best knowledge, this is the first reported case of Eales’ disease that presented with neovascular glaucoma in the first visit. Herein, we describe the clinical presentation, laboratory and ancillary tests' findings that might have had some contributions to such an advanced stage at first presentation, as well as the management plan and the response to the treatment.

## Case presentation

A 24-year-old male from India presented to the emergency room with a one-day history of a sudden decrease in vision in the left eye. He also complained of pain and redness in the involved eye for the last three weeks along with a frontal headache. However, he denied any history of nausea, vomiting, cough, fever, night sweats, change in appetite, change in weight, joint swelling or skin rash. The patient had no history of contact with a known case of tuberculosis or sick patients and did not have any recent travel to his hometown. Also, he is not known to have any history of ocular or systemic diseases such as diabetes mellitus, sickle cell disease or any systemic vasculitis. His surgical and drug history were negative. 

**Figure 1:**
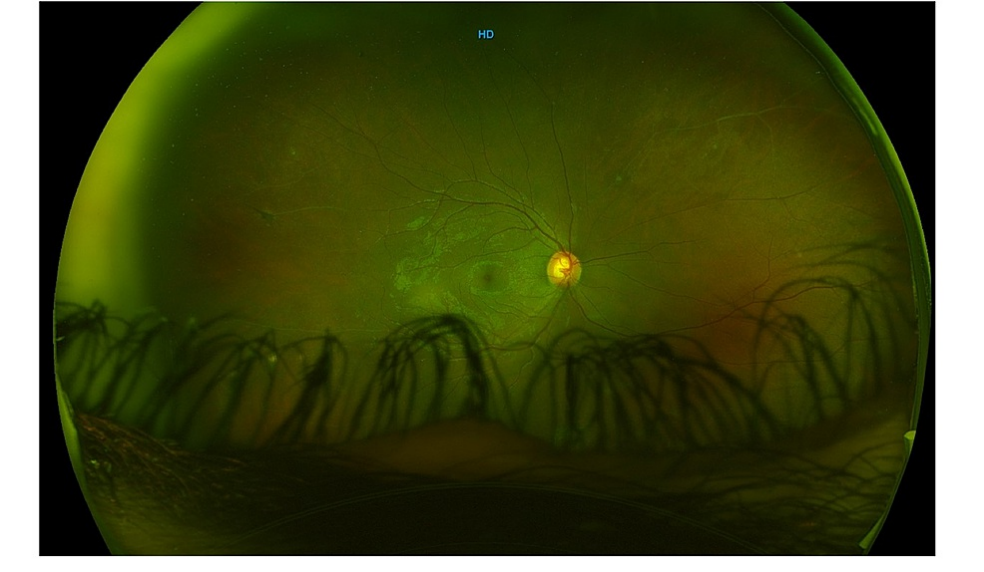
Wide-field image of the right eye showing mid-peripheral vascular sheathing.

**Figure 2:**
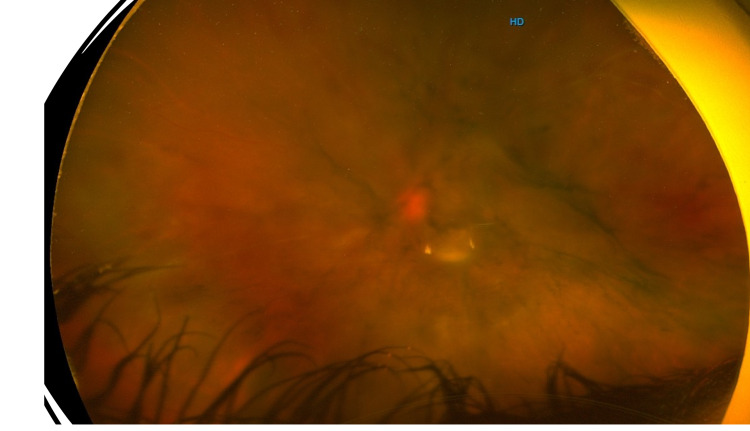
Wide-field image of the left eye showing vascular sheathing with intra-retinal and vitreous hemorrhages.

On examination, his best-corrected visual acuity at the initial presentation was 20/20 in the right eye and counting fingers in the left eye. Slit-lamp examination of the right eye was completely normal. The left eye exam revealed moderate conjunctival injections, micro-cystic corneal edema, clear lens, trace cells in the anterior chamber, 360 iris neovascularization and sluggish slightly dilated left pupil. The intraocular pressure was 11 mmHg in the right eye and 45 mmHg in the left eye. Fundus examination of the right eye revealed vascular sheathing and peripheral ischemic retina with neovascularization, whereas examination of the left eye showed vitreous hemorrhage, intraretinal hemorrhage, extensive retinal ischemia reaching the macula and vascular sheathing (Figures 1, 2). Systemic examination was completely normal.

**Figure 3:**
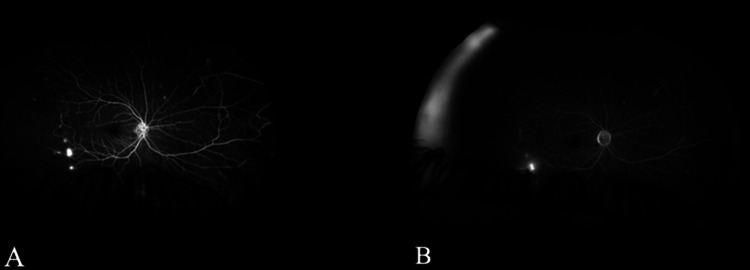
Wide-field angiography of the early (A) and late (B) recirculation phases showing areas of vascular leakage and vascular staining, indicating retinal neovascularization and vasculitis, respectively. The temporal retina is consistently hypo-fluorescent (capillary drop-out), indicating peripheral retinal ischemia.

**Figure 4:**
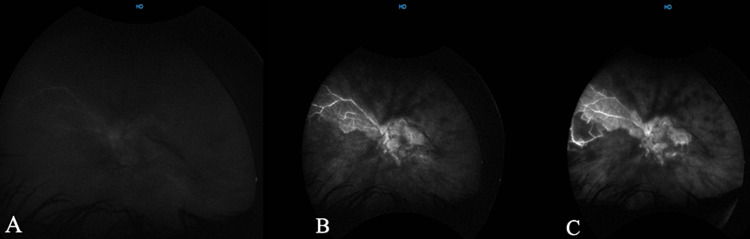
Wide-field angiography of arterial (A) arteriovenous (B) and early recirculation (C) phases showing hypo-fluorescent vessels and a large area of persistent hypo-fluorescence involving the macula, which indicates severe retinal and macular ischemia, along with vascular staining representing vasculitis.

The wide field angiography of the right eye showed mid-peripheral areas of vascular leakage and vascular staining representing retinal neovascularization and vasculitis, respectively (Figures 3A, 3B). The left eye illustrated an extensive area of persistent hypo-fluorescence involving the macula along with vascular staining (Figures 4A-4C).

As Eales’s disease is a diagnosis of exclusion, differential diagnoses of such presentation included proliferative diabetic retinopathy, proliferative sickle cell retinopathy, retinal vein occlusion and retinal vasculitis due to other causes of uveitis. Therefore, further investigations were ordered to exclude the possibility of alternative diagnoses.

The laboratory investigations included complete blood count, fasting blood glucose, erythrocyte sedimentation rate, C- reactive protein level, purified protein derivative (PPD) skin test, antinuclear antibody, syphilis profile, angiotensin-converting enzyme level, sickle cell test and a chest x-ray. All the investigation results were unremarkable except for the PPD test.

The patient was managed immediately in the emergency room with topical and systemic anti-glaucoma, and later, with intravitreal anti-vascular endothelial growth factor (anti-VEGF) injection in the left eye and laser photocoagulation therapy was done to both eyes.

As the PPD test was positive, the patient was managed with anti-tuberculous drugs (isoniazid, rifampin, ethambutol and pyrazinamide) along with systemic steroid at a dose of 1 mg/kg. After two weeks of treatment and close follow-up, the visual acuity of the left eye improved to 20/160 and the intraocular pressure was controlled with topical medications. The patient traveled back to his hometown and lost further follow-ups.

## Discussion

In 1880, Henry Eales first reported Eales’ disease in a group of young males as idiopathic obliterative vasculitis, who had a common history of headache, constipation and epistaxis. The retinal findings included peripheral retinal ischemia, peri-phlebitis and subsequently vitreous hemorrhage [7]. Likewise, this patient presented with frontal headache and vitreous hemorrhage, however, his pain was most likely due to the increase in the intraocular pressure.

Eales’ disease is thought to be multifactorial; however, the most favored etiology is the exposure to tuberculosis and hypersensitivity to tuberculin-protein, as epiretinal membrane samples have been tested positive for Mycobacterium species in previous studies [5,8]. In this report, the PPD test was also positive and indicated previous exposure to Mycobacterium and the possible association with the current ocular presentation.

Diagnosis of Eales' disease is essentially clinical after excluding other possible differentials by history, examination and laboratory tests. The ancillary tests can be performed to delineate complications from retinal ischemia and help in monitoring the disease regression during the treatment [1,5]. Fluorescein angiography for our patient revealed a large area of persistent hypo-fluorescence in the left eye involving the macula, which indicated severe retinal and macular ischemia, along with vascular staining representing vasculitis. This advanced stage of retinal ischemia, as opposed to the usual peripheral retinal ischemia, is thought to be responsible for the neovascular glaucoma being the first presentation.

Therapeutically, the treatment has to be adjusted to the stage of the disease. Systemic medical treatment includes anti-tuberculous drugs and steroids, while ocular interventions involve laser photocoagulation, intravitreal anti-vascular endothelial growth factor therapy and pars plana vitrectomy in advanced stages [9-11]. In this case report, neovascular glaucoma was controlled initially by topical and systemic anti-glaucoma. Laser photocoagulation along with intraocular anti-VEGF injection has led to the regression of the rubeosis iridis and the intraocular pressure was eventually controlled by only topical medications. Systemic prednisolone was also initiated along with anti-tuberculous drugs. In the follow-up visits, the visual acuity improved dramatically.

## Conclusions

Eales' disease is a diagnosis of exclusion and one should ensure a proper history, exam and investigations are conducted to exclude other likely differentials. A possible link to previous exposure to tuberculosis and hypersensitivity to tuberculin-protein has been suggestive, as well as seen in this report. Moreover, extensive retinal ischemia might lead to the unusual presentation of Eales’ disease as neovascular glaucoma.
